# Construction and Evaluation of Nomogram for Hematological Indicators to Predict Pathological Response after Neoadjuvant Chemoradiotherapy in Locally Advanced Rectal Cancer

**DOI:** 10.1007/s12029-022-00861-9

**Published:** 2022-09-14

**Authors:** Keli Wang, Meijiao Li, Jin Yan

**Affiliations:** 1 Department of Clinical Medicine, Southwest Medical University, Luzhou, China; 2 Department of Gastrointestinal Surgery, Sichuan Cancer Hospital & Institute, Cancer Hospital Affiliated to School of Medicine, University of Electronic Science and Technology, Chengdu, China

**Keywords:** Locally advanced rectal cancer, Neoadjuvant chemoradiotherapy, Hematology index, Pathological response, Nomogram

## Abstract

**Objective:**

A retrospective study was conducted by developing prediction models to evaluate the association between hematological indexes, their changes during neoadjuvant chemoradiotherapy (NCRT), and tumor pathological response in patients with locally advanced rectal cancer.

**Methods:**

The clinical data of 202 patients who received NCRT and radical surgery in Sichuan Cancer Hospital were retrospectively analyzed. Univariate and logistic multivariate regression analyses were used to identify hematological indexes with predictive significance. The independent risk factors were imported into the R software, and a nomogram prediction model was developed. The bootstrap method and ROC curve were used to evaluate the discriminative degree of the model.

**Results:**

Univariate analysis demonstrated age, tumor diameter, preoperative T, distance from tumor to the anal verge, CEA before NCRT, preoperative CEA, lymphocyte changes, platelet changes, and pathology of rectal cancer after NCRT were associated. Multivariate analysis demonstrated that age, tumor distance from the anus, preoperative CEA, lymphocyte changes, and platelet changes were independent risk factors. The independent risk factors were imported into the R software to construct a nomogram model. The area under the ROC was 0.76, and the slope of the calibration curve of the nomogram was close to 1.

**Conclusion:**

A low preoperative CEA level, a young age, a high tumor from the anal verge, the maintenance of circulating lymphocyte level, and a decreased platelet level after NCRT are important factors for favorable outcomes after NCRT. Developing a nomogram prediction model with good discrimination and consistency can provide some guidance for predicting pathological responses after NCRT.

## Introduction

The incidence of colorectal cancer worldwide is increasing annually, and according to the 2022 global cancer statistics, colorectal cancer ranks third worldwide in terms of the incidence of malignant tumors and second in terms of mortality [1]. Colorectal cancer is one of the malignant tumors that seriously affects human life and health. Preoperative neoadjuvant chemoradiotherapy can effectively reduce tumor volume, reduce local staging, convert unresectable tumors into resectable tumors, increase the rate of radical resection, and improve the sphincter preservation rate, thereby improving the prognosis of tumor patients [2–4]. Different patients exhibit different pathological responses after neoadjuvant chemoradiotherapy (NCRT). Studies have demonstrated that approximately 7–30% of patients can achieve complete remission (pCR) after NCRT. This pathological complete remission (pCR) recurs in a long distance without the disease. It has a good prognosis in terms of survival and overall survival [5–7]. The characteristics of the tumor itself and the host inflammatory response play an important role in tumor progression, possibly by affecting the tumor microenvironment surrounding rectal cancer, thereby affecting the effect of rectal cancer on NCRT pathological response [8]. Blood cell counts in peripheral blood reflect the tumor microenvironment of patients with rectal cancer [34], so peripheral hematological indicators can be used to evaluate the pathological response after NCRT.

This study aimed to retrospectively evaluate the relationship between peripheral hematological indexes and their changes before and after NCRT in patients with locally advanced rectal cancer and tumor pathological response, to identify hematological indexes that can predict tumor pathological response after NCRT, and establish related predictive models to more effectively guide individualized precision treatment plans for rectal cancer patients.

## Methods

### Patients

We retrospectively collected clinical data from 202 patients with locally advanced rectal cancer who received NCRT at the Sichuan Cancer Hospital between January 2014 and January 2021.

Inclusion criteria: (1) rectal malignant tumor was confirmed by pathological evidence, and the local stage (T3–4 or N +) was confirmed using MRI, CT, rectal ultrasound, and other imaging examinations, and all patients underwent radical surgery after NCRT; (2) aged 18–80 years, gender is unlimited; (3) no anti-tumor therapy before NCRT; (4) no distant metastasis confirmed using imaging evidence; (5) hematological index results before NCRT and before surgery, clinical data complete.

Exclusion criteria: (1) NCRT was not completed; (2) radical surgery for rectal cancer was not performed; (3) emergency surgery was performed because of complications such as tumor perforation and obstruction during NCRT; (4) patients underwent short-course radiotherapy.

### Observation Indicator

General indicators: gender, age, clinical T stage, lymph node metastasis, smoking history, body mass index (BMI), the distance of the tumor from the anal verge (DTAV), maximum tumor diameter, the time interval between the conclusion of NCRT to surgery (weeks), radical surgery, and concurrent chemotherapy.

Hematological indicators: pre-NCRT white blood cell count, lymphocyte count, neutrophil count, red blood cell count, platelet count, anemia, carcinoembryonic antigen (CEA), albumin, granulocyte-to-lymphocyte ratio (NLR), and platelet lymphocyte ratio (PLR); preoperative white blood cell count, lymphocyte count, neutrophil count, red blood cell count, platelet count, anemia, carcinoembryonic antigen (CEA), albumin, NLR, PLR, and PNI; white blood cell changes, neutrophil changes, lymphocyte changes, red blood cell changes, platelet changes between the two.

### Treatment

Neoadjuvant radiotherapy: all patients were treated with conventional fractionated long-course radiotherapy. The radiotherapy technique was intensity-modulated radiotherapy (IMRT) or image-guided radiation therapy (IGRT). The target area was defined as rectal lesions, imaging diagnosis of positive lymph nodes, and pelvic lymphatic drainage area. Gross tumor volume (GTV): the extent of the primary tumor and positive lymph nodes identified by MRI or CT and other imaging examinations; clinical target volume (CTV): combined with the pelvic lymph node drainage area based on GTV, including the rectum and mesangial area, presacral area, internal iliac lymph nodes, and part of the obturator lymph node drainage area. If the tumor invades the pelvic organs, the external iliac lymph node drainage area must also be included; if the tumor invades the anal canal or the lower third of the vagina, additional external iliac and inguinal lymph node drainage areas are required. The total dose of radiotherapy was: GTV and CTV: 50 Gy/25f/2 Gy, 5 days/week or GTV: 50.4 Gy/28f/1.8 Gy, CTV: 45 Gy/25f/1.8 Gy, 5 days/week.

Concurrent chemotherapy: (1) capecitabine: oral capecitabine single-agent concurrent chemotherapy, 825 mg/m^2^ each time, bid, 5 days/week; (2) XELOX: oxaliplatin 135 mg/ m^2^ d1 + capecitabine 1000 mg/m^2^ d1–14; 21 days/time (3) FOLFOX: oxaliplatin 85 mg/m^2^ d1 + fluorouracil 2400 mg/m^2^ for 46 h intravenous infusion + fluorouracil 400 mg/m^2^ d1 + sodium folinate 400 mg/m^2^ d1, 21 days/time.

Radical surgery: all patients underwent TME procedures, including anterior rectal resection and combined abdominoperineal resection.

### Efficacy Assessment

The histopathological regression of the tumor after NCRT was evaluated using the tumor regression grade (TRG) recommended by the American Joint Committee on Cancer (AJCC), 7th edition [11]. TRG 0 (complete remission of tumor): no tumor cells are present in the tissue under a microscope; TRG 1 (near-complete remission of the tumor): single cell or very few small tumor residues in the tissue under a microscope; TRG 2 (partial remission of the tumor): under a microscope, the tumor cells show obvious shrinkage, but the residual tumor cells are more than single or small; TRG 3 (poor or no tumor remission): under a microscope, there is an extensive residual tumor, and the tumor has no obvious shrinkage.

### Data Processing

TRG 0–1 was defined as a good pathological response; TRG 2–3 was defined as a poor pathological response. Male hemoglobin < 120 g/L, female hemoglobin < 110 g/L as anemia; NLR = neutrophil count/lymphocyte count (109/L); PLR = platelet count/lymphocyte count (109/L); blood cell change = pre-NCRT blood cell count − preoperative blood cell count (109/L); CEA change = pre-NCRT CEA − preoperative CEA (ng/ L).

### Statistical Analysis

SPSS 26.0 was used for data analysis, measurement data were expressed as *x* ± s, the *t*-test was used for normally distributed data, and the Mann–Whitney *U* test was used for skewed distribution data; *χ*^2^ test or Fisher’s exact test was used for comparison between groups. The receiver operating characteristic (ROC) curve was used to determine the NLR, PLR, PNI, and CEA before NCRT and NLR, PLR, PNI, and CEA before NCRT, as well as changes in white blood cells, lymphocytes, and neutrophils. The optimal cut-off value of red blood cell change and platelet change was divided into high and low groups. First, univariate analysis was performed on clinical factors, and logistic regression analysis was performed on variables with *P* < 0.05 in univariate analysis to evaluate their relationship with the pathological response. *P* < 0.05 indicates a statistically significant difference. The screened independent risk factors were used to establish a nomogram prediction model with the R software. The calibration curve was obtained by the Bootstrap method, and then the C-index was calculated. The nomogram prediction model was validated by drawing the ROC curve and calculating the area under the curve (AUC) to evaluate the predictive performance of the risk model.

## Results

Analysis of overall characteristics: a total of 202 patients were included in this study, including 158 males and 44 females; the average age was 54.47 ± 10.73 years; 59 patients underwent radical resection of abdominal perineum combined with rectal cancer; 143 patients underwent anterior rectal resection. The tumor length diameter was 5.25 ± 2.20 cm, and the average distance between the lower edge of the tumor and the anal verge was 5.28 ± 2.27 cm; preoperative imaging assessment was performed in 96 patients with T3 stage, 106 with T4 stage, and 168 with lymph node metastasis assessed through imaging, whereas 34 cases without lymph nodes. The mean interval between NCRT and surgery was 10.99 ± 5.72 weeks; 38 cases (18.8%) had TRG 0 points after NCRT; 42 (20.8%) had TRG 1 points; 92 (45.5%) had TRG 2 points); 30 (14.9%) had TRG 3 points.

Univariate analysis of general clinical characteristics: no significant differences in gender, BMI, smoking history, lymph node metastasis, surgical method, concurrent chemotherapy regimen, and pathological response after NCRT for locally advanced rectal cancer (all *P* > 0.05). Age (*P* < 0.001), tumor diameter (*P* = 0.02), preoperative T stage (*P* = 0.043), and distance from the lower edge of the tumor to the anal verge (DTAV) (*P* = 0.029) were statistically significant (Table 1).

**Table 1:** Univariate analysis of clinical characteristics

Characteristics	Number	Efficacy evaluation		*χ*^2^	*P*
		Good response	Poor response		
General situation					
Gender				0.247	0.619
Male	158	64	94		
Female	44	16	28		
Age				14.365	< 0.001
> 50	128	38	90		
≤ 50	74	42	32		
Smoking history				0.213	0.644
Yes	100	38	62		
No	102	42	60		
BMI (kg/m^2^)				0.80	0.777
> 24	68	26	42		
≤ 24	134	54	80		
Maximum tumor diameter (cm)				5.445	0.02
> 3	167	60	107		
≤ 3	35	20	15		
Preoperative T-stage				4.090	0.043
T3	106	49	57		
T4	96	31	65		
Preoperative N-stage				0.348	0.555
N +	168	65	103		
	34	15	19		
Distance from anus (cm)				4.76	0.029
> 4	128	58	70		
≤ 4	74	22	52		
Operation				0.08	0.774
AR	143	61	82		
APR	59	19	40		
Interval time				3.164	0.075
> 8 weeks	150	54	96		
≤ 8 weeks	52	26	26		
Chemotherapy				0.10	0.995
Capecitabine	36	14	22		
XELOX	141	56	85		
FOLFOX	25	10	15		

Univariate analysis of hematological indicators before NCRT: the white blood cell count, lymphocyte count, and red blood cell count before NCRT. The neutrophil count, platelet count, albumin, whether or not anemia, NLR, PLR, and the pathological response of locally advanced rectal cancer after NCRT were not statistically significant (*P* > 0.05), whereas serum CEA level was significantly associated with locally advanced rectal cancer. The pathological response of rectal cancer after NCRT was statistically significant (*P* = 0.005, Table 2).

**Table 2:** Univariate analysis of hematological indexes before NCRT

Characteristics	Number	Efficacy evaluation		*χ*^2^	*P*
		Good response	Poor response		
WBC		6.44 ± 1.98	6.57 ± 1.85		0.740
Neutrophils		4.27 ± 1.68	4.34 ± 1.54		0.973
Lymphocyte		1.55 ± 0.63	1.64 ± 0.55		0.166
RBC		4.52 ± 0.58	4.45 ± 0.53		0.404
Platelet		226.86 ± 80.85	223.18 ± 79.71		0.811
Anemia				3.040	0.081
No	168	62	106		
Yes	34	18	16		
NLR				1.807	0.187
> 2.53	112	49	63		
≤ 2.53	90	31	59		
PLR				0.014	0.905
> 147.9	94	42	52		
≤ 147.9	108	38	70		
CEA				8.056	0.005
> 8.2	55	13	42		
≤ 8.2	147	67	80		
Albumin				3.388	0.066
> 40	150	65	85		
≤ 40	52	15	37		

Univariate variable analysis of preoperative hematological indicators: white blood cell count, lymphocyte count, neutrophil count, red blood cell count, platelet count, anemia, NLR, PLR, and pathological response after neoadjuvant therapy for rectal cancer were not statistically significant (both *P* > 0.05). The serum CEA level was significantly associated with the pathological response of locally advanced rectal cancer after NCRT (*P* < 0.001, Table 3).

**Table 3:** Univariate analysis of preoperative hematological indexes

Characteristics	Number	Efficacy evaluation		*χ*^2^	*P*
		Good response	Poor response		
WBC		4.01 ± 1.45	4.10 ± 1.45		0.515
Neutrophils		2.77 ± 1.21	2.84 ± 1.20		0.5
Lymphocyte		0.76 ± 0.55	0.70 ± 0.26		0.937
RBC		3.89 ± 0.48	3.85 ± 0.42		0.781
Platelet		154.49 ± 49.25	164.53 ± 51.51		0.215
Anemia				0.112	0.738
No	154	60	94		
Yes	48	20	28		
NLR				2.691	0.101
> 2.86	156	57	99		
≤ 2.86	46	23	23		
PLR				3.857	0.050
> 175	142	50	92		
≤ 175	60	30	30		
CEA				14.046	< 0.001
> 1.5	123	36	87		
≤ 1.5	79	44	35		
Albumin				0.713	0.389
> 40	137	57	80		
≤ 40	65	23	42		

Univariate analysis of the changes in hematological indexes: the changes in white blood cells, neutrophils, red blood cells, and the pathological response of rectal cancer after neoadjuvant therapy were not statistically significant (*P* > 0.05), and the changes of lymphocytes (*P* = 0.001), platelet changes (*P* = 0.005), and CEA changes (*P* = 0.013) were significantly associated with pathological responses after neoadjuvant therapy for rectal cancer (Table 4).

**Table 4:** Univariate analysis of hematological index changes

Characteristics	Number	Efficacy evaluation		*χ*^2^	*P*
		Good response	Poor response		
Change of WBC				1.698	0.193
> 3.16	61	20	41		
≤ 3.16	141	60	81		
Change of neutrophils				2.057	0.151
> 1.13	111	39	72		
≤ 1.13	91	41	50		
Change of lymphocyte				11.904	0.001
> 0.585	148	48	100		
≤ 0.585	54	32	22		
Change of RBC				2.411	0.121
> 0.605	100	45	55		
≤ 0.605	102	35	67		
Change of platelet				8.053	0.005
> 47.5	109	53	56		
≤ 47.5	93	27	66		
Change of CEA				6.178	0.013
> 6.9	49	12.00	37.00		
≤ 6.9	153	68.00	85.00		

The clinical indicators with statistical significance in the univariate analysis, such as age, tumor length, tumor distance from the anus, T stage, CEA level before NCRT, preoperative CEA level, lymphocyte changes, platelet changes, and CEA changes, were included in the logistic multivariate regression analysis. The regression analysis results were as follows: age (*P* = 0.003, OR = 0.352, 95% CI: 0.176–0.703), distance from tumor to the anus (DTAV) (*P* = 0.037, OR = 2.113, 95% CI: 1.047–4.266), preoperative CEA (*P* = 0.005, OR = 0.372, 95% CI: 0.187–0.74), changes in *lymphocytes (P* = 0.002, OR = 0.297, 95% CI: 0.136–0.648), changes in platelets (*P* = 0.045, OR = 2.016, 95% CI: 1.015–4.004) was an independent risk factor affecting the pathological response of locally advanced rectal cancer after NCRT (Table 5).

**Table 5:** Logistic multivariate regression analysis of variables

Factors	*B*	SE	Wald	*P*	OR	95%CI
Maximum tumor diameter	− 0.751	0.437	2.956	0.086	0.472	0.2–1.111
Preoperative T-stage	− 0.445	0.343	1.68	0.195	0.641	0.327–1.256
Age	− 1.045	0.353	8.757	0.003	0.352	0.176–0.703
Distance from anus (cm)	0.748	0.358	4.36	0.037	2.113	1.047–4.266
CEA (before NCRT)	− 1.794	1.203	2.225	0.136	0.166	0.016–1.757
CEA (after NCRT)	− 0.989	0.351	7.936	0.005	0.372	0.187–0.74
Change of platelet	0.701	0.35	4.011	0.045	2.016	1.015–4.004
Change of lymphocyte	− 1.213	0.398	9.306	0.002	0.297	0.136–0.648
Change of CEA	1.198	1.248	0.922	0.337	3.314	0.287–38.261

**Fig. 1:**
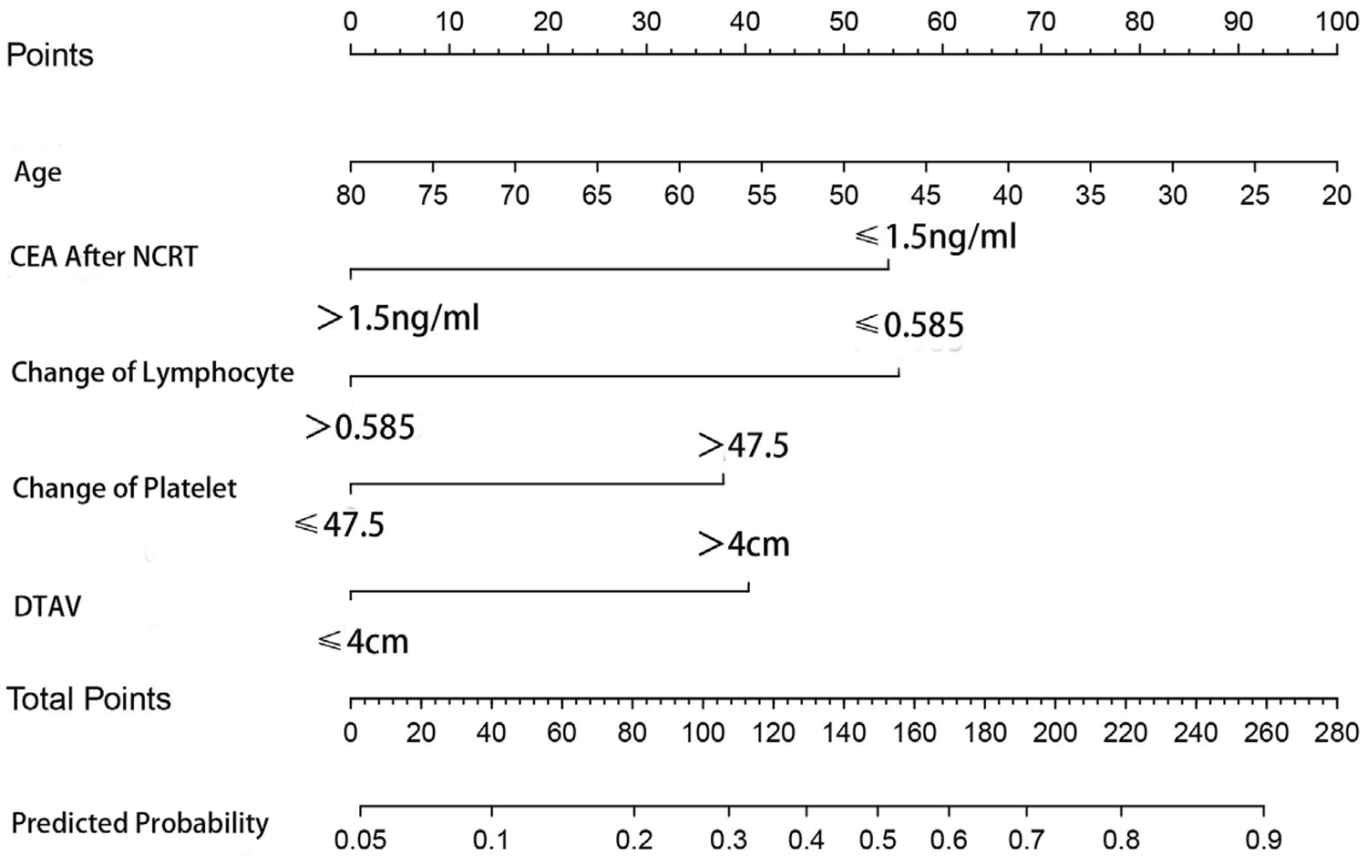
Nomogram model for predicting pathological response after NCRT

**Fig. 2:**
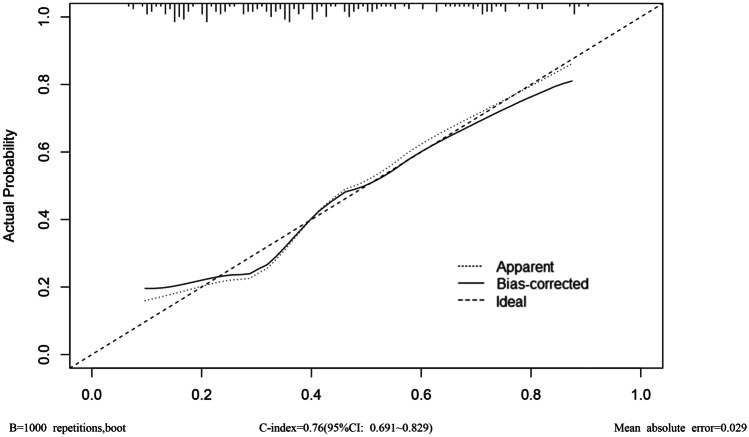
Calibration curve of the nomogram

**Fig. 3:**
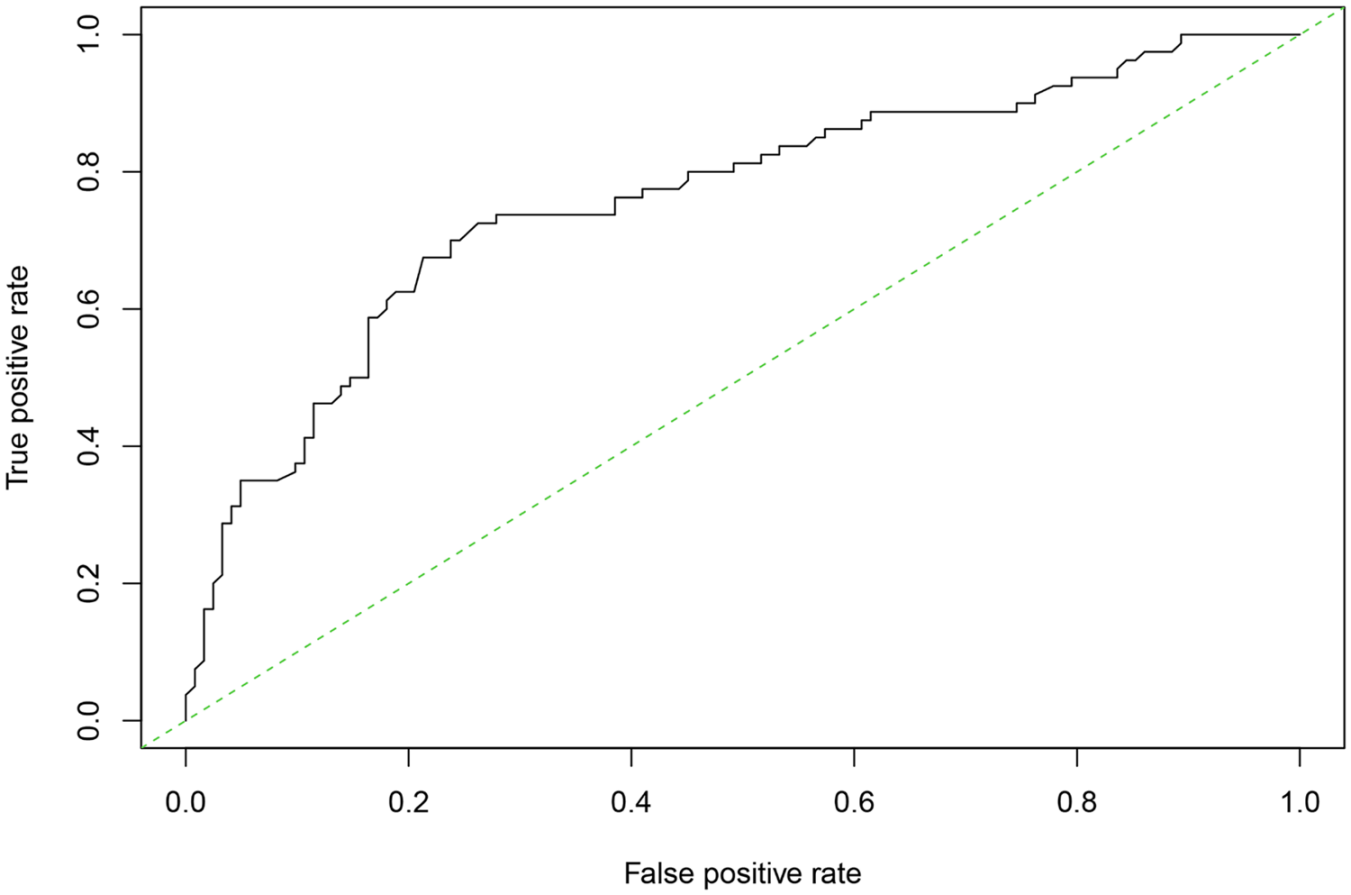
ROC curve of nomogram model predicting pathological response after NCRT

Model development and prediction effect analysis: the predicted factors screened out in the multi-factor logistic regression were imported into R software to construct a nomogram prediction model (Fig. 1). The model assigns a score to each risk factor, and the value corresponding to the total score is the predicted probability of a good pathological response after NCRT. This model was internally validated using bootstrap self-sampling and computing the discriminativeness of predictive model. Through bootstrap repeated sampling 1000 times, the calibration curve of model was obtained (Fig. 2), demonstrating that the nomogram model had good consistency between the predicted probability of occurrence of good pathological response and the actual probability of occurrence after NCRT for rectal cancer. The calculated C-index value was 0.76 (95%CI: 0.691 to approximately 0.829), which means that the discriminative ability of the nomogram prediction model is good. By drawing ROC curve (Fig. 3), the results indicated that the AUC of ROC curve of the nomogram prediction model was 0.76 (95%CI: 0.691 to approximately 0.829). Thereby, the prediction model has good prediction performance and discrimination ability.

## Discussion

Numerous studies have demonstrated that the pathological response of rectal cancer after NCRT is dependent on factors such as tumor differentiation, tumor T stage, tumor distance from the anus, CEA level before NCRT, and the time interval between NCRT and operations [9–12]. Our study demonstrated that age, tumor distance from the anus, CEA levels before surgery, lymphocyte changes, and platelet changes were independent risk factors for the pathological response after NCRT. Studies have revealed [13] that younger patients are more likely to obtain a good pathological response, consistent with our findings, because younger patients may have a stronger immune response to NCRT. However, several studies [14, 15] revealed no significant statistical significance between age and pathological response after NCRT, and even Leow’s [16] study indicated that young age was a predictor of a lower pCR rate after NCRT because young patients may have more aggressive pathological features, associated with higher CD133 + cancer stem cell burden, thereby resulting in a poorer response to neoadjuvant therapy. Further investigation is needed on the pathological response of age to NCRT. Several studies [17, 18] have demonstrated that the distance from the tumor inferior border to the anal verge (DTAV) can be used as a predictor of pCR, but the optimal cutoff value is still unclear. Patel et al. [17] investigated 827 cases of rectal cancer and confirmed that DTAV is an independent risk factor for pCR. The author revealed that 30% were 8 cm tumors, 17% were 8–10 cm tumors, and 14% were tumors larger than 10 cm (*P* = 0.002). However, our study used 4 cm as the distinguishing criterion, and our results revealed that DTAV larger than 4 cm obtained better good pathology. The probability of response was higher (OR = 2.092, 95% CI: 1.046–4.183), consistent with previous results. However, other studies used 5 cm as the boundary between low and high rectal cancer. Peng [19] and other studies used 5 cm as the standard; they observed no statistical significance in pCR after DTAV and NCRT (*P* = 0.464).

Carcinoembryonic antigen (CEA) is a tumor-associated antigen with important clinical value in the status monitoring and efficacy evaluation of colorectal cancer. Studies have believed that a low level of CEA before treatment is an important predictor of good tumor response, but the CEA criticality criteria remain controversial. A study of 531 locally advanced rectal cancer patients with NCRT by Yang et al. [11] revealed that CEA ≤ 5 ng/mL before neoadjuvant therapy was associated with pCR (*p* = 0.021), and a low level of CEA before NCRT was a predictor of pCR (OR = 0.435, 95 m^2^ CIx: 0.214–1.010, *P* = 0.03). Both Lee et al. [20] and Wallin et al. [21] confirmed that low CEA (≤ 5 ng/mL) is a predictor of good pathological response after NCRT. In a study of 218 LARC patients, Li et al. [22] determined 3.35 and 7.48 ng/mL with the maximum cut-off value of ROC analysis as the cut-off value of CEA to predict pCR and good pathological response after NCRT, and multivariate analysis indicated that both CEA ≤ 3.35 ng/mL can predict pCR (OR = 1.427, 95% CI: 1.192–1.709, *P* < 0.001) and CEA ≤ 7.48 ng/mL can predict a good pathological response after NCRT (OR = 1.022; 95% CI: 1.006–1.039; *p* = 0.007). However, a study by Kalady et al. [23] indicated that CEA ≤ 2.5 ng/mL before treatment was not associated with pCR (*P* = 0.21). In our study, the mean CEA of the pathological response group was 6.21 ± 8.15, and the mean CEA of the poor response group was 11.30 ± 18.48. We screened out 8.2 ng/mL by ROC analysis as the best cut-off value for CEA before NCRT (sensitivity 0.838, specificity 0.344), and univariate analysis revealed that CEA ≤ 8.2 ng/mL was associated with the pathological response after NCRT (*P* = 0.005), but there was no statistical significance in multivariate analysis (*P* = 0.136, OR = 0.166, 95% CI: 0.016–1.757).

Some studies have demonstrated that CEA after NCRT can predict the pathological response after NCRT. Peng [24] and other studies indicated that CEA ≤ 2 ng/mL can be used as a predictor of pCR (OR = 1.579, 95% CI: 1.026–2.432; *P* = 0.038); Kleiman [25] and other studies revealed that the CEA level was significantly reduced after NCRT in pCR patients (1.7 vs. 2.4 ng/mL, *p* = 0.003). In multivariate logistic regression analysis, low CEA level after NCRT was an independent predictor of pCR (OR = 1.74, 95% CI: 1.06–3.81); Saito et al. [26] revealed that serum CEA level after NCRT was lower than 5 ng/mL for patients with higher tumor shrinkage rates than those with serum CEA levels ≥ 5 ng/mL. In our study, the mean CEA of the good preoperative pathological response group was 2.02 ± 1.64, and the mean CEA of the poor response group was 3.69 ± 7.16. We screened out 1.5 ng/mL by ROC analysis as the best cut-off value of preoperative CEA (sensitivity 0.55, specificity 0.713), and univariate analysis demonstrated that CEA ≤ 1.5 ng/mL was associated with the pathological response after NCRT (*P* < 0.001), and multivariate analysis revealed that low level of CEA was a predictor of pathological response after NCRT (*P* = 0.005, OR = 0.372, 95% CI: 0.187–0.74).

Some studies have indicated that the change of CEA before and after NCRT predicts pathological response. A study on the change of CEA level on the pathological response of NCRT by Hu et al. [27] demonstrated that the decrease of CEA level was an independent predictor of pCR (training set: OR = 8.25, 95% CI: 2.19–31.10, *P* = 0.002; validation set: OR = 8.30, 95% CI: 1.56–44.17, *P* = 0.013). Kleiman et al. [25] also demonstrated that the normalization of CEA after NCRT strongly predicts good pathological response (OR = 64.8, 95% CI: 2.53–18,371). In our study, ROC analysis considered 6.9 ng/mL as the cutoff standard (sensitivity 0.85, specificity 0.303), and the results revealed that CEA changes were associated with pathological response (*P* = 0.013) but could not be used as a predictor (OR = 3.314, 95% CI: 0.287–38.261, *P* = 0.337).

Host immune and inflammatory responses to malignant tumors are important factors in the occurrence, progression, treatment, and prognosis of various cancers [28, 29]. Studies have confirmed that NCRT can lead to tumors through direct cytotoxic and cytostatic effects. Recent research has suggested that NCRT can induce antitumor immune responses, leading to tumor regression [30]. Lymphocytes represent effector cells that are the main components of the body’s anti-tumor immunity and inhibit cancer cell proliferation and metastatic spread [31, 32]. Studies have noted a sharp drop in circulating lymphocyte counts during NCRT, which is considered a risk factor for poor tumor prognosis. Liu et al. [33] revealed that maintaining a high level of lymphocyte count during NCRT was associated with improved pathological response and survival after neoadjuvant therapy after LARC. Kitayama et al. [34] demonstrated the possibility that circulating lymphocytes may have significant biological effects on tumor response to NCRT. Radiation-induced suppression of circulating lymphocytes may reduce the likelihood of pathological remission after NCRT by allowing regrowth through the proliferation of tumor cells surviving after radiation injury, thereby suggesting that a more pronounced decrease in peripheral blood lymphocytes may be associated with adverse pathological responses. This is consistent with the best cut-off value of 0.585 (10^9^/L) for ROC analysis in our study (sensitivity 0.4, specificity 0.82), and the results demonstrated that the decreased number of lymphocytes after radiotherapy was associated with a good pathological response (*P* = 0.002, OR = 0.297, 95% CI: 0.136–0.648), consistent with previous studies.

Platelets have a wide range of functions, including adhesion, coagulation, and promotion of angiogenesis; they are not only involved in hemostasis but also in the entire inflammatory response [35], and they also play an active role in tumor progression and metastasis. Platelet plays a key role in tumor progression and metastasis through various mechanisms by promoting the occurrence, adhesion, proliferation, chemotaxis, and metastasis of malignant tumors [36, 37]. Elevated platelet counts are a negative predictive and prognostic marker of pathological response in locally advanced rectal cancer undergoing NCRT [38]. Kawai  et al. [39] indicated that platelets might play a key role in regulating the resistance of colorectal cancer to radiotherapy. A retrospective study of 965 cases by Belluco et al. [40] also confirmed that low platelet count before NCRT was an independent positive predictor of pCR. In our study, the platelet count before NCRT (*P* = 0.811) and the platelet count after NCRT (*P* = 0.215) were not statistically significant. The best cutoff value was 47.5 (10^9^/L) in the platelet change in the ROC analysis (sensitivity 0.4, specificity 0.82), and the results revealed that thrombocytopenia was more associated with a good pathological response after NCRT (*P* = 0.045, OR = 2.016, 95% CI: 1.015–4.004), consistent with previous results.

Clinically, we frequently use neutrophil/lymphocyte (NLR) versus platelet/lymphocyte (PLR) to assess a patient's systemic inflammatory response, the neutrophil to lymphocyte ratio (NLR), which can reflect the tumor-promoting balanced relationship between immune response and antitumor immune response [41]. However, the prediction of NCRT response by NLR and PLR in locally advanced rectal cancer is still controversial. In a study of 176 patients with rectal cancer, Kim et al. [41] suggested that good tumor pathological response was associated with pre-NCRT NLR < 2.0 (OR = 2.490, 95% CI: 1.264–4.904, *p* = 0.008); a systematic review and meta-analysis demonstrated that patients with rectal cancer and low NLR who received neoadjuvant radiotherapy had an increased likelihood of pCR (OR = 2.01, 95% CI: 1.14–3.55, *p* = 0.02) [42]. No significant relationship existed between tumor degradation or pathological response after chemoradiotherapy. A retrospective study of 202 cases of rectal cancer by Shen et al. [43] demonstrated that NLR < 3 was not statistically significant between pCR and non-pCR groups (65.8% vs. 69.3%, respectively; *P* = 0.674). The optimal cut-off value of NLR remains unclear. Hodek et al. [31] and other studies considered NLR from 1.8 to 4.2 every 0.2 as a critical value to evaluate the relationship between NCRT and pCR, and the results demonstrated that all critical values could not predict pCR (*P* > 0.05). In our study, the best cut-off value of ROC analysis was used as the cut-off standard, and the results demonstrated that neither of them was statistically significant (*P* = 0.187, *P* = 0.101), which may be related to the cut-off standard of NLR, and more research is required to evaluate the relationship between NLR and pathological response after NCRT.

Currently, the role of PLR in predicting pathological response after NCRT in locally advanced rectal cancer is still controversial. A retrospective study of 291 cases of rectal cancer by Lee et al. [44] indicated that higher PLR after preoperative chemoradiotherapy was significantly associated with poor tumor response. Kim et al. [41] demonstrated that PLR < 133.4 before NCRT was associated with good tumor response (OR = 3.009, 95% CI: 1.477–6.127, *p* < 0.001). In a retrospective study including 297 LARC patients, Lee et al. [45] indicated that for locally advanced rectal cancer, high PLR and PLR changes during NCRT are important predictors of pCR, and the degree of PLR increased during treatment is the most accurate predictor of pCR. However, several studies have demonstrated no significant relationship between PLR and tumor degradation or pathological response after chemoradiotherapy for rectal cancer [31, 43]. Our study used the best cut-off value of ROC analysis as the cut-off standard. The cut-off value of PLR before NCRT was 147.9 (sensitivity 0.525, specificity 0.574), and the cut-off value of preoperative PLR was 175 (sensitivity 0.375, specificity 0.754), which revealed that PLR before NCRT was not related to pathological response (*P* = 0.905), whereas PLR and pathological response after NCRT was *P* = 0.05. Although there was no statistical significance, the results may indicate that low PLR is related to good pathological response (35.5% vs. 50%). The current demarcation criteria for PLR require more studies to evaluate the relationship between PLR and pathological response after NCRT.

We developed a nomogram prediction model based on the independent risk factors screened using multivariate logistic regression. Through the established model, we can evaluate the pathological response after NCRT. Compared with the single risk factor analysis, our model combined several statistically significant risk factors, scored each, and assessed the patient's pathological response through a combination of different risk factors. The models evaluated by the bootstrap method and ROC curve have good discrimination and consistency, providing guidelines for the pathological response of locally advanced rectal cancer after NCRT.

Our study is a single-center retrospective study; there may be selection bias, the established model has not been validated by external data, the study sample size is small, and the only hematological indicators in our study are from the pre-NCRT and preoperative data. Metrics were analyzed without continuous analysis of changes in hematologic metrics during NCRT.

## Conclusions

The pathological response was better in younger patients; the tumor’s inferior margin was further from the anal margin, the preoperative CEA level was low, and NCRT maintained the circulating lymphocyte count while decreasing the platelet count. The development of a nomogram prediction model has good discrimination and consistency and can provide certain guidelines for predicting pathological response after NCRT for locally advanced rectal cancer.

## Data Availability

The datasets used and analyzed during the current study are available from the corresponding author on reasonable request.
